# Towards inclusive social appraisal: risk, participation and democracy in governance of synthetic biology

**DOI:** 10.1186/s12919-018-0111-3

**Published:** 2018-07-19

**Authors:** Andrew Stirling, K. R. Hayes, Jason Delborne

**Affiliations:** 1 Science Policy Research Unit, University of Sussex, Falmer, Brighton, BN1 9RH UK; 2 Data61, CSIRO, GPO Box 1538, Hobart, TAS 7001 Australia; 3 Department of Forestry and Environmental Resources, North Carolina State University, 2820 Faucette Dr, Raleigh, NC 27695 USA

## Abstract

Frameworks that govern the development and application of novel products, such as the products of synthetic biology, should involve all those who are interested or potentially affected by the products. The governance arrangements for novel products should also provide a democratic mechanism that allows affected parties to express their opinions on the direction that innovation does or does not take. In this paper we examine rationales, obstacles and opportunities for public participation in governance of novel synthetic biology products. Our analysis addresses issues such as uncertainties, the considering of alternative innovations, and broader social and environmental implications. The crucial issues in play go beyond safety alone, to include contending social values around diverse notions of benefit and harm. The paper highlights the need for more inclusive social appraisal mechanisms to inform governance of Synthetic Biology and alternative products, and discusses a few practical methods to help achieve this goal.

## Background

New synthetic biology [1] and gene drive [2] technologies raise prospects like: altering or suppressing entire populations of disease vectors or agricultural pests by releasing just a few genetically modified organisms [3]; or revolutionising agricultural production by synthesising products such as palm oil [4]. These developments have led to widespread reconsideration of current risk-based governance mechanisms as means to balance associated risks and opportunities [5]. Here we define governance to be the set of regulatory processes, mechanisms and organizations through which political actors influence environmental actions and outcomes [6]. Risk-based mechanisms aim to help the decision maker balance the potential benefits of innovation against the potential harms to humans and their environments, whilst recognising the uncertainties associated with both [7].

Current reconsiderations within the field of risk assessment emphasise that the assessments should not be restricted in scope to human safety-related parameters but should embrace wider ecological and societal issues [8], which will likely raise deeper questions about the governance of synthetic biology in democratic societies [9–11]. Other pertinent issues in this view include the need for methods to help formalise the inherently subjective choices that determine how a risk-assessment is framed [12], characterise the quality of quantitative information used in a risk assessment [13], and how to make governance procedures flexible and precautionary in face of the “deep uncertainty” that accompanies many new technologies [12].

The desire to include deliberative procedures within the governance arrangement of synthetic biology has led various agencies to assert the need to engage communities, stakeholders and broader publics in decision making processes. The National Academies of Sciences, Engineering and Medicine [13] for example, recommends that “defined mechanisms and avenues for [public] engagement should be built into the risk assessment and decision-making process from the beginning”. This recommendation is echoed by the International Risk Governance Council [14] who call for regulatory agencies to “prototype new approaches for iterative risk analyses that incorporate external peer review and public participation”.

It implies no necessary criticism of such calls to note that public participation, as opposed to communication, is not routinely practised in existing risk assessments, and important questions can be raised about underlying rationales. In the words of a former executive in the US Environmental Protection Agency, there typically exist highly diverse and often contending motivations to enact procedures nominally referred to as “participatory” [15]. Aims may variously be: ‘substantive’ - i.e. about making better decisions; ‘normative’ – i.e. about pursuing appropriate process in a democracy; or ‘instrumental’ - about engineering pre-existing aims [16]. This latter category includes: fostering trust [17]; providing justification [18]; securing acceptance [19]; and managing blame [20]. In this paper we focus on substantive approaches to public participation that involve genuine empowerment of all affected parties in the interests of making better choices among contending innovation or policy pathways in any given field [21].

It is also important to recognise that these drivers for more participatory practices are not new. Similar calls for more inclusive risk-based governance have been made previously in the context of genetically modified plants [22], and for risk assessment more generally [23], on the stated grounds that: (i) early public engagement can provide information that improves decisions; (ii) including stakeholders and the public in the decision-making process leads to more trusted decisions; and, (iii) citizens have a right to influence decisions about issues that affect them [24]. If public engagement exercises around synthetic biology or gene drives are to be credible or robust in the substantive terms described above, then they should not be restricted to issues of risk or safety alone, nor confined merely to the ways in which a new technology should be introduced [25].

Rather than focussing on one particular innovation, participatory practices need to provide a balanced analysis of the relative pros and cons of a diversity of contrasting policies and innovations that are able to address the same societal or environmental functions [21]. This prevents the process being reduced simply to a means to modulate implementation of one particular technology. Comparative appraisal opens the possibility that a strongly-backed new technology might, under appropriate circumstances or perspectives, be deprioritised in favour of an alternative strategy [21]. As highlighted in a recent report for the UK Government Chief Scientist, the enabling of societies to exercise agency over the directions taken by innovation in this way is, at one level, a basic imperative of democracy [26].

It is against this background that this paper examines the opportunities and barriers that exist to substantive public engagement and participation in risk-based governance frameworks. A key emphasis will lie in exploring relations with quantitative, probabilistic approaches to risk assessment that have recently been recommended for synthetic biology products like gene drives. We pay attention to examining mechanisms that assist substantive public engagement and so allow for more societally robust and democratically accountable social choices of technology in these fields [27].

The issues addressed in this paper, then, are not just about whether or what kind of public participation might be required in each step in a typical risk assessment. Instead, it is risk assessment itself that is placed in wider context. Nor is this simply a matter of ‘bolting on’ some additional processes around a conventional regulatory appraisal. Since the answers obtained in risk assessment depend on both questions and assumptions, the point becomes clear that if risk assessment itself is to be regarded as rigorous, then it needs to be as systematic and robust about its own qualitative framing conditions as it already tries to be about quantitative data and analysis [28].

So, public participation should not be seen as a matter of ‘political correctness’ or as a means to achieve a pre-conceived end, but rather as inherent to the rigour and effectiveness of regulatory assessment. It allows attention to extend beyond crucial questions over ‘how safe?’, to address equally imperative issues over ‘which way?’ innovation should go in any given field; and ‘who says?’ and ‘why?’ [28]. If these substantive kinds of issue are attended to, then regulatory assessment of synthetic biology and gene drives can move from a purely risk-based analysis, to diverse – more substantive – processes of ‘social appraisal’ [17].

## Risk assessment and social appraisal

**Fig. 1:**
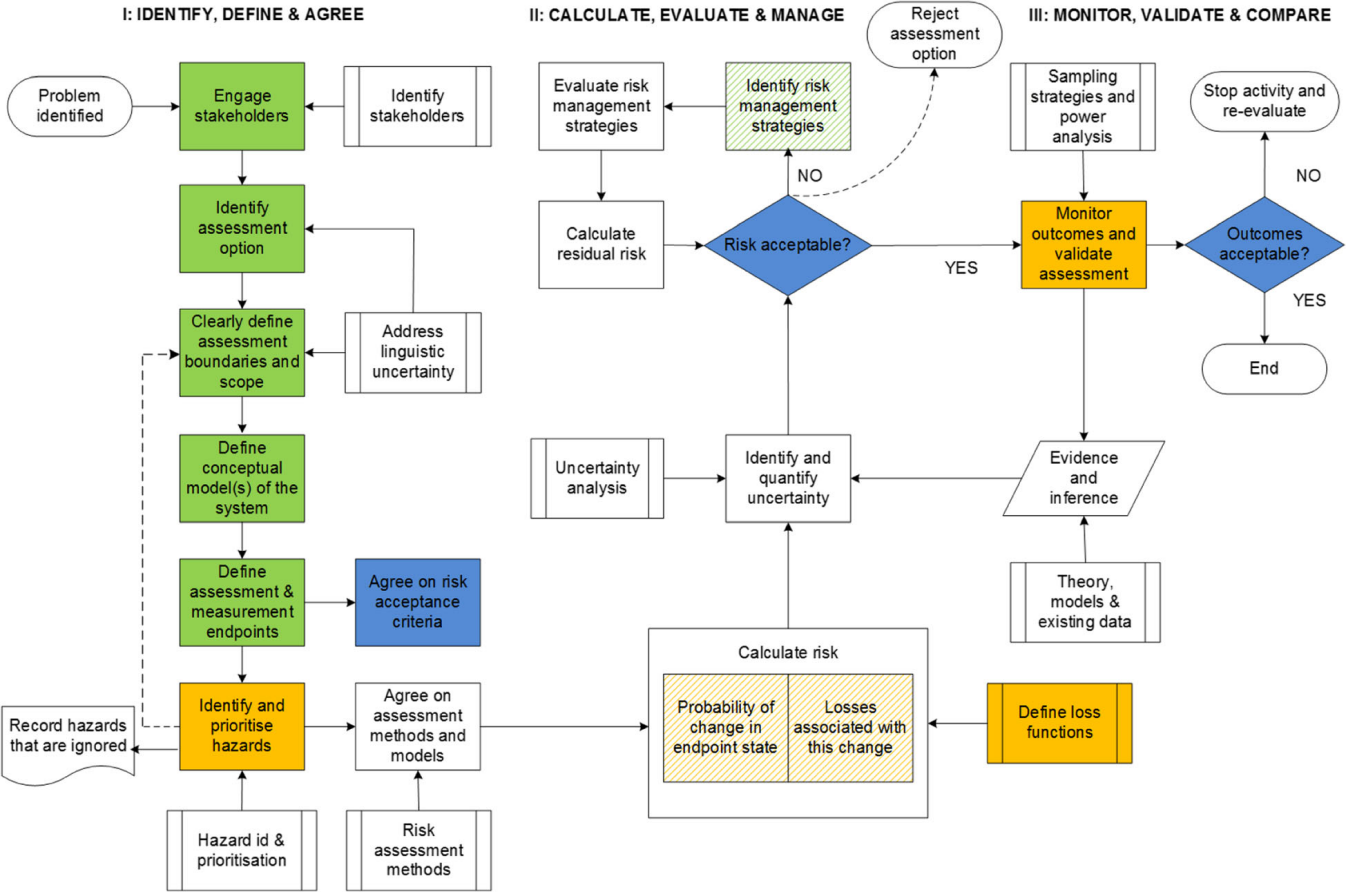
An idealised scientific risk assessment process (amended from [29]). A flow chart showing steps where public participation would be essential under a social appraisal process and easily facilitated (solid green), essential but harder to achieve (solid orange), and essential but difficult to achieve (solid blue) for novel synthetic biology products. Pattern shading represents steps where public participation may be useful but not essential to a social appraisal process

In this section, we consider the practical implications of the issues raised above for a risk-based governance system of synthetic biology products such as gene drives. To this end, Fig. 1 kicks off with an overview of the steps involved in a conventional risk assessment process. It is important to emphasise that this Figure is a simplified and idealised version of a risk assessment process. In practise, risk assessments rarely if ever follow all the stages, or pursue the sequence described here with precision. It is presented here as a model to facilitate the discussion within the paper.

The assessment has essentially three stages, labelled: (i) Identify, define and agree; (ii) Calculate, evaluate and manage; and (iii) Monitor, validate and compare. The steps within each of these three stages were originally described in the context of assessing and managing the environmental risks posed by the release of transgenic fish [29]. We have annotated these steps to distinguish those parts of the overall risk assessment process where public participation is essential if the assessment is to broaden and achieve the substantive imperative of a social appraisal process.

To begin, it is widely recognised to be essential, at least for novel technologies such as gene drives, that ‘the public’ are engaged in the opening steps of the first (identify, define and agree) stage of a risk assessment process. Indeed, these first steps are designed to engage the public in an initial discussion about the problem that the technology or product is designed to address, and the extent to which this problem might be addressed with other new or existing technologies or practices.

Public engagement at this stage is essential for several reasons. In the first instance, different ‘publics’, that is constituencies with contrasting understandings, values and interests, must agree that the problem at hand is real and that a novel technological solution is at least worth contemplating. Failure to successfully engage diverse publics at this stage is likely to lead to immediate opposition, as occurred with Oxitec’s proposal to release genetically engineered sterile male mosquitoes in the Florida Keys [30]. Furthermore, any activity that potentially exposes publics to environmental or health risks is unethical if there are alternative, more benign options, or if there is no expectation of possible benefits [31]. In other words, there must be some agreed basis that a problem currently exists, leading to appropriate justification of need [32].

If a particular technological ‘solution’, such as a synthetic gene drive, is propounded as a potentially effective response to an agreed problem, either on its own or as part of an integrated package of solutions, then additional community engagement is desirable for ethical and practical reasons [33]. In terms of the risk analysis steps, stakeholders can at this point make useful contributions to: (i) defining the boundaries and scope of the assessment, for instance concerning which alternatives are considered; (ii) describing conceptual models of the environmental and socio-economic systems that the options will interact with; (iii) identifying valued components or processes of these systems (assets); and (iv) identifying circumstances that could lead to adverse outcomes (hazards) if the technology is deployed. Facilitated discussions with broad groups of stakeholders at this stage have been shown to improve the conceptual understanding of systems and the hazard identification stage [34].

In these early stages of the process it is particularly important that the governance regime does not restrict or suppress an adequate exploration of alternative (social or technological) responses. Appraisal should devote symmetrical attention to all considered alternatives and offer a balanced picture of associated pros and cons as seen by affected stakeholders – particularly those that have no commercial interest in the technology concerned. For instance, many effective alternative innovations in seed production are often excluded from regulatory appraisal processes around the world, in favour of more energetically-propounded transgenic options that offer attractive private benefits from intellectual property, profits from value chains or sales of associated products [24]. These neglected alternatives can include: ‘marker selection’ [35]; participatory breeding [36]; agricultural extension services [37]; and open source seed sharing methods [38], which all harness the innovative capacities of farmers themselves and help tailor crop development to local conditions [39]. If alternative approaches like these are to be given a fair hearing, then they must be addressed by wider practices of social appraisal that extend risk assessment attention beyond a single or narrow set of options.

The third main component in Fig. 1 - identifying assets - becomes particularly important when addressing novel technologies, because the ‘assets’ determine assessment endpoints – i.e. values that risk assessment is trying to protect. The assessment endpoints in turn determine the measurement endpoints – i.e. the things that the risk assessment will make predictions about [40], and these should be used to identify the risk acceptance criteria that support a decision and a compliance monitoring strategy. Public engagement is essential at this step, in order to ensure that the risk endpoints, and hence acceptance criteria, reflect the values that affected communities actually hold. If risk assessment fails to do this, the focal product is unlikely to be considered acceptable, irrespective of the assessment outcomes. If assessment is not understood to address community values, it is likely to be perceived as irrelevant, at best – or at worst, as representing other vested interests.

Practically it is more difficult to undertake public engagement in the second stage in the idealised framework (labelled Calculate, evaluate and manage in Fig. 1). This stage aims to determine risks for an agreed set of priority hazards. Risk calculations can be performed qualitatively or quantitatively, but here we focus on probabilistic risk assessment calculations. With a probabilistic risk assessment, the scope for public engagement in this second stage is much reduced, largely because of the particular kinds of expertise required and associated barriers around styles of knowledge and understanding including language (discussed below).

Probabilistic risk assessment for novel technologies, at least initially, must rely on opinions and beliefs. Classical actuarial approaches are not possible because the technology’s operational history is limited and/or its potential adverse outcomes occur at a very low frequency. A key feature of probabilistic risk assessment in these circumstances is that opinions are typically elicited from experts using formal methods (see for example [41] carefully designed to minimise the various forms of ambiguity caused by the natural vagueness of language [42, 43]), and to provide predictions that can be (in)validated with observations. In theory these methods could be employed to elicit the opinions of stakeholders and thereby help to expose differences in opinion that might be masked by different interpretations of the same word, such as “negligible”. But we are unaware of any examples of this in practise, perhaps because of the barrier that probabilistic methods present.

It is still possible, but somewhat more difficult, to engage publics around the formulation of what in risk assessment parlance are termed ‘loss functions’. These functions express the loss that occurs following a predicted change in the value of a measurement endpoint – i.e. they measure the possible consequences should adverse outcomes occur. In financial contexts, loss functions are typically expressed as a change in the monetary value of a portfolio over time [44]. In a human health context, loss is also relatively readily defined [45, 46]. Such functions can help make explicit the particular value judgements embedded in any given assessment that underlie a specific interpretation of impact, and facilitate comparisons with other equally-reasonable values that might yield different interpretations [47, 48]*.* In ecological contexts, the concept of loss is more ambiguous and value laden – so it is therefore more desirable, but at the same time more difficult, for publics to be engaged in the formulation of the way in which the ecological consequences of adverse outcomes are measures and expressed [49–51].

It is also essential for interested and affected communities to be engaged in this second stage of the risk assessment procedure around issues of “acceptability”. For new technologies this is typically a difficult stage. Public engagement is only meaningful here if involved communities have previously contributed to risk acceptance criteria (stage 1) and have also been kept informed of observed outcomes, and decisions that arise following these observations (stage 3). Perhaps most importantly, a focus on “acceptance” can only be considered valid, if the assessment gives equal attention to the pros and cons of a variety of alternative technologies or strategies with the same policy aims.

Finally, the overall objective of the third and last stage of the conventional risk assessment framework is to compare risk predictions to observed outcomes. Testing risk and benefit predictions against observed outcomes is an important science quality criteria: to comply with the scientific method, risk predictions must be, at least theoretically, capable of being invalidated by observations. At this stage it is also possible – and sometimes desirable on cost grounds – to engage interested and affected communities in monitoring strategies through, for example, citizen science activities [52].

Again, in substantive terms, it is important to emphasise that questions around benefit and harm must be directed to the potential pros and cons associated with a diverse array of alternative policy options. It should be noted, however, that contrasting dimensions of each option may not necessarily be subject to simple trade-offs, and methods beyond those usually associated with risk-based governance mechanisms may be needed to address the complex, dynamic and uncertain relations between wider social and environmental values – as well as entirely-valid and reasonable, but non-utilitarian ways of reasoning [53, 54].

**Fig. 2:**
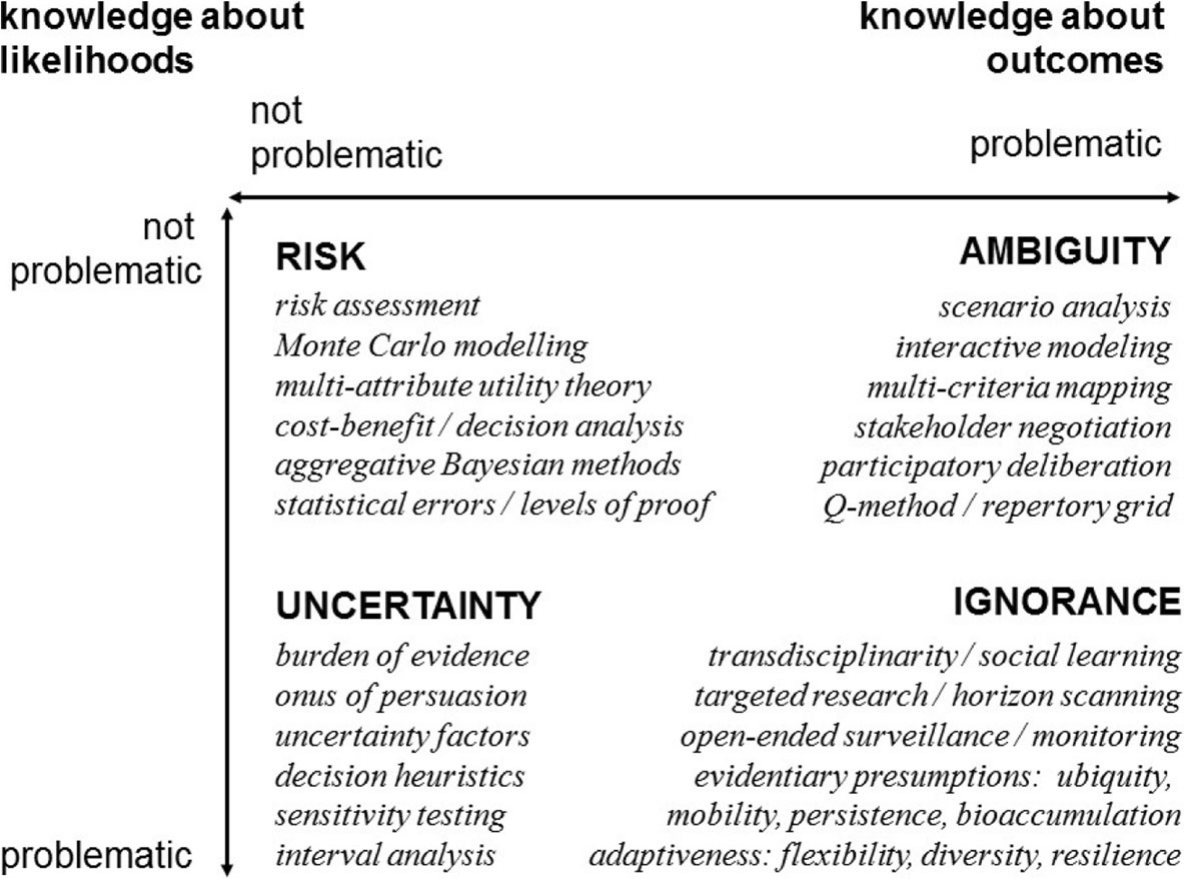
Different aspects of incertitude. As distinguished in relation to the fundamental parameters of risk assessment (probabilities and outcomes) (adapted from [28])

For example Fig. 2 shows one characterisation, among many other variants in the literature [55–58] of four contrasting aspects of incertitude. This identifies a variety of methods that may serve useful functions in substantive social appraisal – including as bridges, catalysts and frameworks for wider processes of public participation. Terminology can be controversial, so the point of Fig. 2 is not to insist on words. The term ‘uncertainty’, for instance, is used in a variety of sometimes opposing ways [59, 60]. Bayesians assert that subjective probability is the only coherent way to perform the types of comparisons across multiple options with uncertain outcomes that a substantive social appraisal process demands [61] and that subjective probability is in principle an adequate way to represent uncertainty due to knowledge gaps and the uncertainty caused by the inherent variability of many real-world processes [62].

Other probability theorists point out, however, that it must be acknowledged at least in principle, that there is always a possibility that a situation will arise under which there exists no firm basis for confidence in the values that might be taken by probability distributions [63]. Indeed, under these conditions, some probability theorists acknowledge a condition under which – in objective terms – probabilities simply “do not exist” [64]. This may be because neither historical evidence nor the completeness of available models are felt to be sufficient to derive likelihoods for all relevant real-world outcomes. To assert single probability distributions under such conditions would involve “pretence of knowledge” [65]. Following longstanding usage in policy appraisal over the past century [66, 67], it is this condition that is referred to in Fig. 2 here as ‘uncertainty’. Other terms may legitimately be preferred, but it is crucial not to let reluctance to name this condition lead to a situation in which it is simply ignored [28].

The practical point here, however, is clear. The key issues that arise are: (i) probabilistic methods can present a barrier to public participation; and, (ii) it is crucial to consider whether alternative approaches to uncertainty provide a coherent and transparent way to analyse pros and cons of different technological solutions. It is to this end, that frameworks like that offered in Fig. 2 can offer one useful input among many, in prompting greater consideration.

A crucial further point in Fig. 2 is that challenges in social appraisal do not just involve problematic knowledge about likelihoods, but also different kinds of ‘contradictory certainty’ over meaning [68]. Referred to as ‘ambiguity’ – meaning the condition of being open to more than one interpretation - in Fig. 2, these disagreements may concern: interests or values; ‘benefits’ or ‘harms’; or alternative policy options [69]. It is a matter of analytical rigour to recognise that such dilemmas also mean that there can in principle exist no uniquely optimal analytical solution [70, 71]. This further underscores the substantive importance of participation [72–75].

Taken together, the main issue that arises in all this is simply the need to recognise: a) that some level of ignorance will always exist with a new technology – where “unknown unknowns” [76] mean “we don’t know what we don’t know” [55]; and b) that a substantive social appraisal entails value based judgements that probabilistic risk assessment techniques are not designed to address. This makes it important that governance embeds risk-based assessment in a broader social appraisal that includes public participation [77].

## Obstacles and promises of public participation

What the preceding discussion has shown is that the scope and methods of risk-based governance need to be broadened if the substantive issues raised by new technologies like synthetic biology and gene drive technologies are to be addressed. These considerations require attention to a broader set of practical methods, beyond those currently used in risk assessment, for wider social appraisal [78]. Here, a multiplicity of forms of public participation become recognisable as crucial means to help achieve both analytical rigour and democratic accountability in the framing and implementation of governance measures.

Potential obstacles to public participation in the crucial first steps of the first stage of a risk assessment include language barriers, conflicting styles of knowledge, and availability and accessibility of information. The extent to which these are actual barriers will vary on a case by case basis, but in general these obstacles are likely to be more acute in developing nations [78].

Cost is also a significant barrier – from the perspectives of both sponsors and participants. Public participation activities can be costly to organize, in terms of labor, logistics, preparatory materials, and design of interactions [79]. Equally costly is the time and effort expended by participants, especially in a political-cultural environment that does not make it clear that such participation will make a meaningful difference in decision making [80]. Put another way, a major obstacle is the credibility that it will be worthwhile for publics to bother to participate.

Under a view that participation will focus primarily on modalities for implementation, and participation is merely about ‘deciding how to do it’, the value of the engagement is the sharing of perspectives and knowledge, and such sharing can be costly. For example, the costs of organizing engagement at each step of a laboratory study would be prohibitive, suggesting that some balance needs to be achieved in terms of the frequency and intensity of engagement and its costs to enact.

The transaction costs of public engagement are more difficult to compute and less relevant, however, under a contrasting view that participation should extend to the possibility that an alternative strategy will be substituted. Here the value of engagement could be larger, but harder to determine, if the costs of adverse effects are avoided by pursuing an alternative strategy.

Either way, we note that “transaction costs” implies that what is valuable is the exchanged “material,” while the transaction itself is worthless and should be minimized to the greatest extent for the sake of efficiency. In the case of engagement, however, the transaction has value - both in terms of what is exchanged and the experience of connecting with others who have different knowledge, perspectives, and values. These transactions have the potential to build relationships, trust, and insight. The value of these achievements are difficult to measure but we nonetheless suggest it is helpful to consider both the “transaction costs” and “transaction benefits” of stakeholder and public engagement.

Synthetic biology products and gene drives in particular may raise specific challenges in this context: (i) they are often described in very technical, domain-specific, terminology that is not accessible to a wide audience. To understand their production processes and modes of action requires a high degree of training or a significant amount of editorial effort to de-mystify the language; (ii) techniques such as CRISPR based gene-editing have lowered technological barriers and substantially compressed design to production cycles, enabling the field to move rapidly. This in turn can reduce the lead-in time for risk assessment and social engagement activity; and, (iii) low threshold gene drives will theoretically spread throughout the domain of an entire target population. If a target species has a large range, for example a mosquito malaria vector found across sub-Saharan Africa, the number and diversity of potential stakeholders could be larger than that encountered with other new products. The nature and severity of these issues will vary on a case by case basis, but taken together they could significantly raise the costs of a substantiative engagement process.

On the other hand, of course, costs incurred when unduly narrow governance circumscribes assessment, excludes alternatives or sidelines relevant uncertainties can prove to be very large [81, 82]. Once realised, it can be extremely costly to address (potentially irreversible) environmental effects [83, 84] or shift away from technologies that have already locked in [26, 85–87]. These possible wider burdens of narrow governance are important to take seriously, because they often fall most heavily on people who are in other ways most excluded and vulnerable [21, 88–90].

Against this background, there are a diversity of practical participatory methods. For instance, risk assessment may usefully be informed by carefully-structured workshops like Problem Formulation Options Assessment (PFOA) methodology [91]. The PFOA methodology has been specifically designed to front-end a risk-based governance framework for novel technologies and has been successfully applied to GM crops in Kenya [92]. Multicriteria mapping is another approach that has been applied with some success [93]. Also yielding concrete quantitative pictures – alongside a rich body of qualitative information – concerning a diversity of contrasting innovation pathways, this has also been used to explore GM maize and parallel options in Kenya [94].

Engaging stakeholders in the conceptual modelling and hazard identification stage of the risk assessment can be facilitated by using graphical conceptual modelling methods, such as cartoons, influence diagrams and Signed Directed Graphs [95]. Graphical conceptual methods also provide a structure for, and therefore facilitate, public participation in the identification of assessment and measurement endpoints, and can also guide the development of quantitative models that are otherwise difficult to engage the public in due to the technical hurdles that they present. Signed Directed Graphs have added advantages in this context as they provide the basis for analysis of the effects of feedback in complex systems [96], including the socio-economic systems that are coupled to, and drivers for, the environmental systems that may be perturbed by a novel technology.

Irrespective of any particular methods, substantive aims in technology governance should also be responsive to ‘uninvited’ engagement by marginal voices on their own terms [97]. Many methods also exist to help enable this – including: open space [98], participatory rural appraisal [99], deliberative mapping [100], do-it-yourself juries [101], participatory technology assessment (https://ecastnetwork.org), and action research [102] – which if properly undertaken can help to further these aims.

Despite the complexity and diversity of views, then, it is possible to draw some firm overall conclusions. Quite simply, broader public participation in environmental decision making leads to better quality decisions [103]. There exists much scope for, as well as obstacles to, practical extensions of existing procedures in order to enable meaningful participatory deliberation.

In the end, the most important questions in this process are not just about ‘yes or no?’, ‘how much?’ or ‘how fast?’ concerning a circumscribed partisan selection of possible ‘solutions’ – but rather about fundamental issues for democracy over: ‘which way?’, ‘who says?’ and ‘why?’ Addressing such questions – in collaborations that span domains of expertise and civil society – can ‘open up’ a diversity of alternative viable policy responses [104]. How societies address the uncertain benefits and risks of these alternative responses, however, remains a contentious issue and one that requires much more attention than this paper allows.
